# Advances in Genetic Characterization and Genotype–Phenotype Correlation of Duchenne and Becker Muscular Dystrophy in the Personalized Medicine Era

**DOI:** 10.3390/jpm10030111

**Published:** 2020-09-03

**Authors:** Omar Sheikh, Toshifumi Yokota

**Affiliations:** 1 Department of Medical Genetics, University of Alberta Faculty of Medicine and Dentistry, Edmonton, AB T6G 2H7, Canada; osheikh1@ualberta.ca; 2 The Friends of Garrett Cumming Research & Muscular Dystrophy Canada HM Toupin Neurological Science Research Chair, Edmonton, AB T6G 2H7, Canada

**Keywords:** Duchenne muscular dystrophy (DMD), exon-skipping therapies, next-generation sequencing (NGS), Sanger sequencing, multiplex ligation probe amplification (MLPA), multiplex polymerase chain reaction (PCR), comparative genomic hybridization array (CGH), viltolarsen, eteplirsen, golodirsen

## Abstract

Currently, Duchenne muscular dystrophy (DMD) and the related condition Becker muscular dystrophy (BMD) can be usually diagnosed using physical examination and genetic testing. While BMD features partially functional dystrophin protein due to in-frame mutations, DMD largely features no dystrophin production because of out-of-frame mutations. However, BMD can feature a range of phenotypes from mild to borderline DMD, indicating a complex genotype–phenotype relationship. Despite two mutational hot spots in dystrophin, mutations can arise across the gene. The use of multiplex ligation amplification (MLPA) can easily assess the copy number of all exons, while next-generation sequencing (NGS) can uncover novel or confirm hard-to-detect mutations. Exon-skipping therapy, which targets specific regions of the dystrophin gene based on a patient’s mutation, is an especially prominent example of personalized medicine for DMD. To maximize the benefit of exon-skipping therapies, accurate genetic diagnosis and characterization including genotype–phenotype correlation studies are becoming increasingly important. In this article, we present the recent progress in the collection of mutational data and optimization of exon-skipping therapy for DMD/BMD.

## 1. Introduction

Duchenne muscular dystrophy (DMD), a severe neuromuscular disorder, affects the skeletal and cardiac muscle of 1 in 5000 newborn boys [1], with very few treatment options available [2]. Frame-shifting mutations in the dystrophin gene [3] cause DMD by removing production of the 427 kDa protein dystrophin [4]. Without dystrophin, progressive muscle wasting occurs [5]. By contrast, in-frame dystrophin deletion mutations lead to the related condition Becker muscular dystrophy (BMD), which ranges in phenotype from subclinical to borderline DMD [6]. For this reason, the term DBMD is used to indicate the range of conditions that arise from dystrophin mutations. Though most mutations reported fit the dystrophin reading frame rule stated above, there are a significant number of exceptions, highlighting an intricate genotype–phenotype relationship [7]. Given the range of mutations underlying DBMD, precise genetic diagnosis and genotype–phenotype correlation analysis are crucial to design mutation-specific therapeutics like exon skipping [8,9]. Box 1 describes the keywords used in this article. A better understanding of genotype–phenotype relationships in DBMD patients may lead to better design of exon-skipping therapies [9]. In this review, we describe the recent advances in molecular diagnostic approaches for DMD/BMD and discuss how exon-skipping therapy can be optimized.

Box 1 **Definitions of keywords used in this article.**

**Genotype**: genes that encode physical characteristics of an organism.
**Phenotype**: the observed characteristics resulting from the expression of those genes.
**Intron**: non-coding region of DNA that is removed by splicing prior to translation.
**Exon**: coding region of gene that appears in the mature RNA transcript.
**In-frame mutation**: a mutation that does not disrupt the reading frame of a gene during the transcription, likely not interfering with protein production.
**Out-of-frame mutation** (also known as frameshift mutation): a mutation that disrupts the reading frame, likely destroying protein production.

## 2. Sequencing and Genetic Diagnosis Methodologies Relevant to DBMD

Sequencing and mutation detection strategies are intertwined in DBMD. The key strategies used to study this disorder are listed below in Table 1.

Sequencing methodologies provide precise genetic testing that can clarify the mutations seen in patients. Sanger sequencing is performed using nucleotides that lack a 3′-hydroxyl group, preventing the DNA polymerase from continuing the DNA chain at that position [17]. Though low throughput, it can complete partial sequencing cheaply.

Southern blot was originally used to examine DMD mutations before other techniques replaced it. Southern blot analysis using cDNA probes, which were established earlier [18], has been used to detect deletions and duplications of the dystrophin gene [19,20,21]. Southern blotting, however, is no longer commonly employed for DMD since it is time-consuming and requires several hybridization steps [11].

The use of multiplex polymerase chain reaction (PCR) for mutation detection has played a more prominent role in DMD genetic diagnosis [13]. Multiplex PCR, which allows for rapid detection of mutations using small or suboptimal samples of genomic DNA, is more efficient than Southern blotting [13]. One study indicated that the majority of the deletions detected by use of cDNA probes and Southern blot in the study could have been also characterized by multiplex PCR [20]. In 2006, Stockley et al. established the use of quantitative multiplex PCR to screen all 79 exons for deletions and duplications [15], strengthening the technique’s applicability in DMD.

Multiplex ligation-dependent probe amplification (MLPA) acts well as a first-pass assessment of DMD due to its speed and cheap cost [22]. This technique detects exon deletions and duplications. An MLPA probe consists of two probe oligonucleotides that hybridize to adjacent sites of the target sequence, followed by probe ligation. Probes hybridized are amplified by PCR and quantified, providing amplification products of unique size. The MLPA approach then provides the relative copy number of target sequences [22], which can detect most of the deletions and duplications in the DMD gene.

One rising alternative to MLPA is comparative genomic hybridization (CGH) array [16]. Since 2004, this approach has marked a new milestone for genetic diagnosis [23]. CGH is performed by using probes covering dystrophin exons and introns conjugated to a glass slide. Control and patient DNA is fragmented and hybridized to the probes, allowing for the detection of the relative abundance of each exon. However, unlike MLPA, it can pinpoint the location of breakpoints within introns [16]. This method can be applied to screen the genome both at the whole-gene level and the individual exon level for many disease genes including DMD [24]. The CGH platform can detect precise intron breakpoints in high resolution and sensitivity [25] while also being completely scalable [26]. Through the use of CGH, the ability to capture intronic mutations is notably improved [27]. Due to the high resolution of CGH [28], this technique has been used to probe intronic mutations in dystrophin using patient data [29,30]. Therefore, CGH is also a recommended technique used first to look at DBMD genetics.

Next-generation sequencing (NGS), which refers to sequencing strategies featuring a much greater sequencing volume than Sanger sequencing [10], is another prominent strategy relevant to DBMD [11] and can be used alongside other strategies such as MLPA to provide a reliable genetic diagnosis. Overall, targeted NGS can bolster a more precise understanding of ambiguous mutations [12] in contrast to MLPA which cannot identify some dystrophin mutations [11].

NGS features several potential diagnostic uses. For instance, NGS can accurately identify pathogenic small mutations in DBMD patients without a large deletion/duplication, especially in non-coding regions [31]. Therefore, this technique has great potential to improve the molecular diagnosis of DBMD. Lastly, whole-exome sequencing, which solely concentrates on the coding exon regions of the genome, is useful for the quick examination of exonic mutations [32]. Though this technique is not widely used, it has been used to identify small mutations giving rise to DBMD [33,34,35]. The broad range of NGS methodologies available supports precise genetic diagnoses [12].

## 3. Exon-Skipping Therapies for DMD

Exon-skipping therapy is based on the observation that not all of the 79 dystrophin exons are essential for functional protein [36]. Patients with in-frame deletions typically feature a milder BMD phenotype, despite not having all exons, which forms the basis for the approach of exon skipping [37]. Synthetic antisense oligonucleotides (AONs), which are engineered to resist nuclease degradation, are typically used to target mRNA of the dystrophin gene for removal, thereby restoring the reading frame and promoting the production of partially functional protein [36]. This truncated protein then compensates for the function of the full-length protein. Currently, many exon-skipping therapies are in clinical testing [38]. Thus far, exon skipping has shown effectiveness in delaying DMD progression [36]. Eteplirsen, which is designed to skip exon 51 [39], and golodirsen, which is designed to skip exon 53 [40], gained conditional approval in the US in 2016 and 2019, respectively. A newly approved AON, viltolarsen, has been especially promising. Based on compelling evidence of efficacy, viltolarsen received approval in Japan for the skipping of exon 53 [41] and was conditionally approved by the U.S. Food and Drug Administration (FDA) in August 2020 [42]. A Phase II trial of viltolarsen demonstrated that the low dose group (40 mg/kg) rose from an average dystrophin production baseline of 0.3% to 5.7% of normal while the high dose group rose from an average dystrophin production baseline of 0.6% to 5.9% of normal in Western blots [43]. In parallel, the trial strengthened the evidence that viltolarsen can stabilize or improve muscle strength and functionality based on timed tests. Of the three approved therapies, which are compared in Table 2, viltolarsen has produced the highest observed increases in dystrophin production.

For this therapy to effectively treat patients, it must produce a stable dystrophin protein. In one study, researchers examined the stability of edited in-frame dystrophins lacking exons 45–53, exons 46–54, and exon 47–55, respectively; the edited protein lacking exons 46–54 featured the greatest stability [47]. Though this study provides biochemical and computational prediction of exon-skipping therapies, it does not demonstrate these results in vivo [9]. Nevertheless, exon-skipping schemes can cause a myriad of consequences at the protein structure level, which could influence therapeutic effectiveness.

In DMD, exon skipping is still challenged by its mutation-specific nature. Such therapies could be spread too thinly across many different mutations even though it can potentially treat many patients in total. For example, though 47% and 90% of nonsense mutations could be treated using single and double exon-skipping, respectively, this therapy development could necessitate targeting 68 of dystrophin’s 79 exons [48]. Although technically more challenging, double exon skipping substantially raising the applicability of exon-skipping therapies compared to single exon skipping highlights the power of skipping more than one exon. In a dystrophic dog model, double exon skipping of DMD exons six and eight induced by cocktail AONs resulted in the systemic correction of the reading frame and truncated dystrophin expression in skeletal muscles accompanied by improved running speed [49]. The potential of multi-exon skipping is supported by the milder BMD phenotypes observed with the absence of exons 45–55 [50]. In particular, these patients largely featured no mortality and delayed loss of ambulation [51]. Multi-exon skipping of exons 45–55 is expected to benefit 47% of DMD patients [51]. In a DMD mouse model with a deletion mutation in exon 52, exons 45–55 skipping was induced by cocktail AONs, leading to systemic dystrophin expression and functional rescue [52]. Overall, successful development of multi-exon skipping will significantly expand the applicability and optimize the function and stability of truncated dystrophin.

## 4. Patient Registries and the Personalization of Exon Skipping

To better understand which patients are amenable to mutation-specific therapies, including exon-skipping, patient data must be collected broadly through studies and registries. In a foundational study, Baumbach et al. observed that 56% of DMD patients have detectable deletions, 29% of which mapped to a region proximal to the 5’ end of the gene whereas 69% mapped to a region located centrally [53]. The Leiden patient registry reflects one major collection of data on the genetics of DBMD [7]. A large-scale study on the UMD-DMD registry from 2008 was performed on 2405 French patients with DBMD [54]. DMD patients featured 61% large deletions and 13% duplications whereas BMD patients featured 81% large deletions and 6% duplications. Comparatively, this indicates a similar deletion rate to Baumbach et al. Furthermore, this database study indicated that 24% of mutations are de novo events, reinforcing the relatively frequent occurrence of mutations in the dystrophin gene. Finally, this large-scale approach to genotype–phenotype in analysis coincides with the development of other international DMD patients’ registries [54].

TREAT-NMD, an EU-funded multinational network, aims to establish comprehensive information on the natural history of DMD by acquiring data from a large number of patients from a variety of countries not limited to Europe [55]. Currently, the TREAT-NMD database contains a lot of mutational data [56], though as of 2015 15% and 57% of mutations submitted to the registry were from the Americas and Europe, respectively. In parallel, researchers across many countries are collecting mutational data on DBMD patients across the world. These efforts supplement consolidation of patient data into a global registry like TREAT-NMD [57,58,59,60,61,62,63,64,65,66,67].

In 2015, TREAT-NMD’s global database was used to assess more than 7000 dystrophin mutations [56]. Among large mutations, which comprise 80% of total mutations, 86% are deletions and 14% are duplications. This study, beyond providing an overview of mutations observed in a global group of DMD patients, also concludes that the skipping of exons 51 (14% of patients), 45 (9% of patients), 53 (8.1% of patients), and 44 (7.6% of patients) could apply to significant minorities of the registry’s patients.

Inspired by the TREAT-NMD global registry, Japan established its own registry called Remudy. In a 2013 study examining 688 DBMD patients, the deletion of exons was most frequent followed by point mutations and duplications [68,69]. The most recent published analysis of Remudy concluded, based on a set of 1197 Japanese DMD patients, that 107 patients could benefit from exon 51 skipping while 111 could benefit from exon 53 skipping [70].

## 5. Genotype–Phenotype Correlation Studies to Predict the Likely Outcomes of Exon-Skipping Therapies

Through documenting the genotype–phenotype relationship, researchers may better design mutation-specific therapies such as exon-skipping. A greater understanding of genotype–phenotype relationships has been supported by data from clinical studies. Although the reading-frame rule holds in approximately 90% of DBMD cases [7], there are important exceptions. A 2007 review pooled DBMD patient data, based on MLPA, Southern blotting, or PCR analysis, concluded that in-frame deletion patterns result in a mixture of DMD and BMD phenotypes [71]. The deletion of exons 45–47, for instance, featured a 15% occurrence of DMD whereas the deletion of exons 45–51 featured a 48% occurrence of DMD (13 out of 27 patients).

Assessing the genotype–phenotype relationship in a subset of DMD patients might more directly indicate the merits of potential exon-skipping therapies. The 5’ region of the gene, which includes exons 3–9, may be associated with complex genotype–phenotype correlations [72]. In one case study, a patient with an in-frame deletion of exon five featured a more severe than expected BMD phenotype despite the continued recognition of exon six [73]. By contrast, an in-frame deletion of exons 3–9, according to one study, mostly leads to a BMD phenotype [74,75]. The two closely examined patients featured especially mild BMD with only mild heart impairment. In addition, Nakamura et al. reported a patient with this deletion showing only a slight decrease in cardiac function but without muscle involvement at the age of 27 years. By examining this deletion in vivo, the researchers concluded that the removal of exons 3–9 via multi-exon skipping likely generates a mild BMD phenotype. Based on these observations, removal of exons 3–9 is a promising treatment for DMD patients with mutations in this region.

Findlay et al. examined 41 patients enrolled in the United Dystrophinopathy Project focusing on in-frame deletions around exon 45 [8]. All patients with Δ45–46 deletions (n = 4) carried a diagnosis of DMD whereas most patients with Δ45–47 deletions (n = 17) and Δ45–48 deletions (n = 19) were diagnosed with BMD. Based on these findings, the skipping of exon 46 for patients missing exon 45 may not rescue the DMD phenotype. Instead, the study illustrates how the skipping of exons 46–47 or 46–48 for these patients has a greater likelihood of producing a BMD phenotype. As a result of this cohort study, a clinical case can be made for multi-exon skipping, which remains in preclinical testing [76]. From this example, we can see how genotype–phenotype correlations can support the design of exon-skipping therapies, improving their personalization.

A systematic review of dystrophinopathy data from the published literature and unpublished databases examined 135 DBMD patients with in-frame deletions equivalent to the skipping of exon 51 [77]. Of these patients, the majority (n = 81) had BMD whereas 16 patients had more severe phenotypes and 6 had no definitive phenotype. The authors conclude that exon 51 skipping therapy, overall, is likely to produce milder BMD phenotypes in many patients.

To understand the genotype–phenotype relationships of in-frame deletions within the exons 45–55 mutational hot spot, 43 patients with DBMD patients were examined using MLPA, Southern blotting, and multiplex PCR [51]. The deletions examined are as follows: Δ45–55 (n = 7), Δ45–51 (n = 6), Δ45–48 (n = 5), Δ45–57 (n = 3). Researchers subdivided these groups into two groups based on truncated dystrophin conformation: hybrid type (Δ45–55, Δ45–58, Δ45–51) and fractional type (Δ45–57 and Δ45–49). Hybrid type conformation (n = 18) at large features a lower proportion of wheelchair-bound patients than the fractional type conformation (n = 6). Log-rank tests revealed a statistically significant difference between the hybrid and fractional groups (*p* < 0.05) of the age at which patients became wheelchair-bound. In other words, the fractional type appears to more consistently lead to an earlier loss of ambulation. This study provides another manner of predicting the viability of dystrophin protein produced by exon-skipping.

Larger studies of in-frame deletions can more strongly guide exon-skipping development [71]. Looking at in-frame deletions within the hotspot region, researchers determined that some mutations were unexpectedly severe, leading to a DMD phenotype rather than the expected BMD phenotype. For example, in-frame deletions starting from exon 49 and exon 50 featured 92% DMD and 90% DMD proportions, respectively, reinforcing the fact that not all potential exon-skipping strategies will resolve a severe phenotype.

Genotype–phenotype correlations of in-frame deletions also support multi-exon-skipping therapies, especially removing exons 45–55. In three patients each featuring in-frame deletion of the region, two developed heart failure while featuring no overt skeletal pathology whereas a third patient featured muscle atrophy and weakness [78]. The condition of all remained stable with treatment. A separate study examined nine patients with the same mutation and indicated that all nine patients had quadriceps and calf hypertrophy and no respiratory involvement. Meanwhile, two patients featured dilated cardiomyopathy [79]. These results suggest, like with the previous study, that the deletion of exons 45–55 is associated with a milder condition compared to smaller in-frame deletions in this region. A multi-exon-skipping strategy can recapture this phenotype by removing several exons, rather than simply skipping every exon in the region individually, and potentially treat over 65% of DMD patients featuring deletions [80].

## 6. Conclusions

Through comprehensive registries of patient data such as TREAT-NMD with the support of newly available genetic diagnosis tools, DBMD patients can be classified based on mutations, which will further help optimize therapy design while offering higher power for clinical trials [55]. Concurrently, the emergence of multi-exon skipping raises the overall applicability of this treatment strategy, although it is technically more challenging. For exon-skipping therapies to be as effective as possible, cohort studies of genotype–phenotype relationships in DBMD patients with the same resulting mutation would support their design [9]. Because BMD can feature a plethora of truncated dystrophins, exon skipping resulting in truncated dystrophins linked to a milder BMD phenotype might be more beneficial. However, caution should be taken in interpreting these data as other factors, such as the variability of exon skipping efficacy among different exons, also need to be taken into account. Nevertheless, larger cohort studies utilizing patient registry data on genotype–phenotype correlation would greatly contribute to the rational design of mutation-specific therapies including exon skipping in the personalized medicine era.

## Figures and Tables

**Table 1:** Sequencing and genetic diagnosis methodologies relevant to Duchenne/Becker muscular dystrophy (DBMD).

Methodology	Brief Description
Sanger sequencing	Low throughput, conventional strategy with lower cost than more advanced sequencing [10]. Allows for sequencing the dystrophin gene.
Next-generation sequencing (NGS)	Class of more advanced sequencing strategies with high throughput [11]. Can examine whole single genes, panels of multiples genes, all protein-coding genes, or entire genomes [12]. Single gene sequencing is especially powerful in DMD [11].
Quantitative Southern blot	Originally the only reliable method for detecting duplication and identifying carriers [13]; however, this method requires several hybridization steps [14].
Multiplex polymerase chain reaction (PCR)	A strategy that can detect the vast majority of DBMD gene deletions. An improved multiplex PCR assay can detect deletions and duplications in all 79 exons of the DMD gene [15].
Multiplex ligation-dependent probe amplification (MLPA)	A prominent first-pass tool for assessing the genetics of DBMD [11]. MLPA can screen all 79 dystrophin gene exons for deletions and duplications in DBMD patients and carriers but cannot detect most small mutations [12].
Comparative genome hybridization array (CGH)	This tool probes dystrophin exons and introns and can pinpoint the location of intronic breakpoints. CGH is a compelling alternative to MLPA [16].

**Table 2:** Comparison of FDA-approved exon-skipping therapies for Duchenne muscular dystrophy (DMD). Mean dystrophin protein production (as a percentage), relative to healthy controls, is presented based on Western blot data. Baseline values are included for reference.

Therapy	Baseline (% of Normal)	Dystrophin Production (% of Normal)	Side Effects
Eteplirsen [44]	0.08	0.93	No severe or moderate adverse events 8 mild events considered related to treatment [45]
Golodirsen [46]	0.095	1.019	2 moderate adverse events (infection and pyrexia) 8 mild events considered related to treatment
Viltolarsen [43]	0.3 (dose of 40 mg/kg) 0.6 (dose of 80 mg/kg)	5.7 (dose of 40 mg/kg) 5.9 (dose of 80 mg/kg)	No severe or moderate adverse events No mild events considered related to treatment
